# A family of bacterial actin homologs forms a three-stranded tubular structure

**DOI:** 10.1073/pnas.2500913122

**Published:** 2025-03-12

**Authors:** Julien R. C. Bergeron, Shamar L. M. Lale-Farjat, Hanna M. Lewicka, Chloe Parry, Justin M. Kollman

**Affiliations:** ^a^Randall Centre for Cell and Molecular Biophysics, King’s College London, London SE1 1UL, United Kingdom; ^b^Prosemble group LTD, London SE1 1UL, United Kingdom; ^c^Department of Biochemistry, University of Washington, Seattle, WA 98195

**Keywords:** actin, cryo-EM, structural biology, bacterial cytoskeleton

## Abstract

The cytoskeleton is crucial for cell organization and movement. In Eukaryotes, it largely consists of the protein actin, that forms a double-stranded linear filamentous structure in the presence of ATP and disassemble upon ATP hydrolysis. Bacteria also possess actin homologs, that drive fundamental cellular processes, including cell division, shape maintenance, and DNA segregation. Like eukaryotic actin, bacterial actins assemble into dynamic polymers upon ATP binding, however variation in interactions between strands gives rise to striking diversity of filament architectures. Here, we report a family of bacterial actins of unknown function, conserved among the *Verrucomicrobiota* phylum, which assembles into a unique tubular structure in the presence of ATP. A cryo-EM structure of the filaments reveals that it consists of three strands, unlike other described bacterial actin structures. This architecture provides further insights into the organization of actin-like filaments and has implications for understanding the diversity and evolution of the bacterial cytoskeleton.

Actin is a highly conserved protein that plays a crucial role in the structure and function of cells. Actin monomers (G-actin) polymerize to form filaments (F-actin) in the presence of adenosine triphosphate (ATP). Actin also possesses ATPase activity, and the filaments disassemble upon ATP hydrolysis. This activity forms the basis for most cellular processes in Eukaryotic cells, including muscle contraction, cell migration, cell division, intracellular transport, and signal transduction (1).

Bacteria also possess proteins homologous to actin, although each protein performs a specific function: MreB forms short, disconnected filaments that rotate around the cell and help maintain the shape of rod-shaped bacteria (2, 3); FtsA associates with FtsZ to form a ring-like structure at the cell mid-point, known as the Z-ring, and is involved in cell division (4); ParM transport nascent plasmids to the cell poles (5); and MamK anchors magnetosome vesicles along a linear track, ensuring their alignment in the cell (6).

Structural studies have revealed that the overall fold and ATPase activity is similar across actin-like proteins, but they adopt distinct filamentous architectures, proposed to be related to their specific function.

Here, we characterize a previously unknown family of bacterial actin homologs, and show that it forms a three-stranded, polar filament. This unusual architecture suggests a distinct mechanism of filament assembly and enhances our understanding of the evolution of actin-like proteins.

## Results and Discussion

**Fig. 1.**
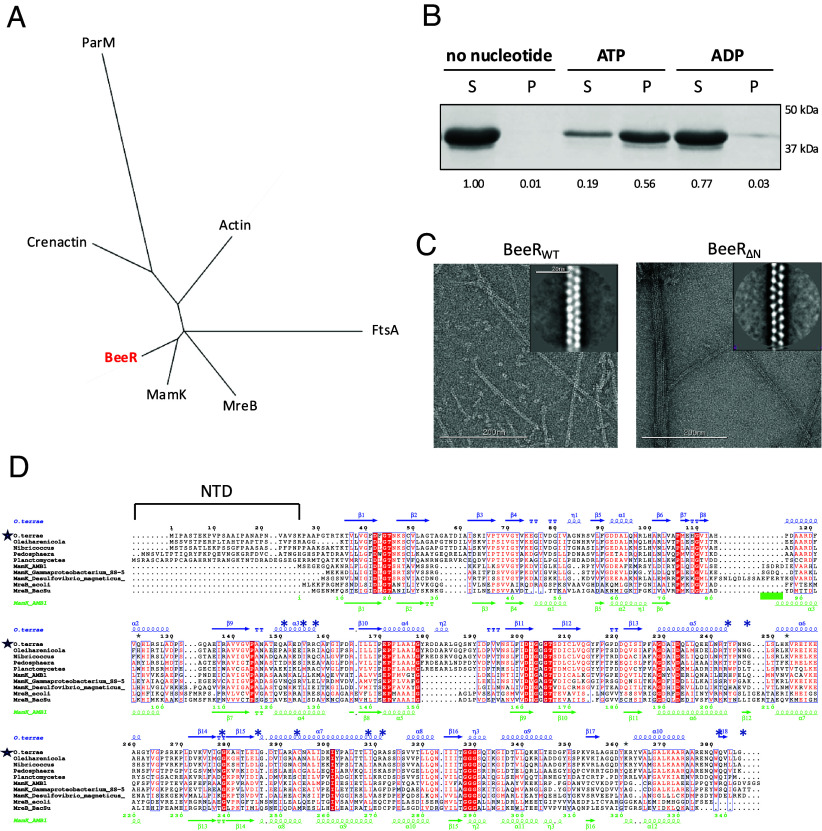
A previously uncharacterized bacterial actin homolog family. (*A*) Unrooted phylogenic tree of the main families of actin ortholog. (*B*) Pelleting assay for BeeR. It is primarily monomeric in the absence of nucleotide, and polymerizes in the presence of ATP, but not ADP. For each sample, the relative band intensity is indicated below. (*C*) Negative-stain TEM micrograph of BeeR filaments obtained with the WT protein (*Left*) and with a construct lacking the N-terminal domain (*Right*). 2D classes of the filaments are shown as inserts. (*D*) Multiple sequence alignment of selected BeeR orthologs, with the *Opitutus terrae* BeeR ortholog indicated with a blue star. The secondary sequence of BeeR and MamK are shown on *Top* (blue) and *Bottom* (green), respectively. The blue * symbol indicates residues located at the cross-strand interface within the three-stranded BeeR filament; the green line indicates the loop forming the cross-strand interface in MamK. The *O. terrae* BeeR ortholog, characterized in this study, is indicated with a blue star.

To identify actin-like proteins that adopt unique filament architectures, we searched for uncharacterized actin homologs in the bacterial metagenomics database. We identified a family of actin homologs, most closely related to MamK (7–10) (25 to 30% sequence identity) (Fig. 1*A*) but present in non-magnetotacic bacteria, and therefore presumably with a distinct function. This protein is highly conserved in the *Verrucomicrobiota* phylum, a family of anaerobic bacteria ubiquitously present in soil samples, as well as in the gut microbiome (11). Sequence analysis confirms that it possesses the conserved residues for ATP binding and hydrolysis (Fig. 1*D*), and structure prediction confirmed that it likely adopts an actin-like fold. One distinctive feature of this family is the presence of an additional 30 to 50 amino acid extension at the N terminus (NTD) (Fig. 1*D*), predicted to be unstructured, and not found in other actin-like proteins.

We next purified one representative ortholog, from the bacterium *O. terrae* (12) (UniProt B1ZZL4). A pelleting assay confirmed that it is soluble in isolation and polymerizes in the presence of ATP, but not ADP (Fig. 1*B*), confirming its actin-like property.

Negative-stain electron microscopy (EM) analysis revealed that these polymers consist of long, well-ordered filaments, which appeared as twisted rail tracks (Fig. 1*C*), up to several μm in length. 2d classification suggested that it adopts a rail tracks-like architecture, consisting of two strands rotating on their individual axis (Fig. 1 *C*, *Inset*). This prompted us to name this family the Bacterial elongated entwined Rail-like protein (BeeR).

**Fig. 2.**
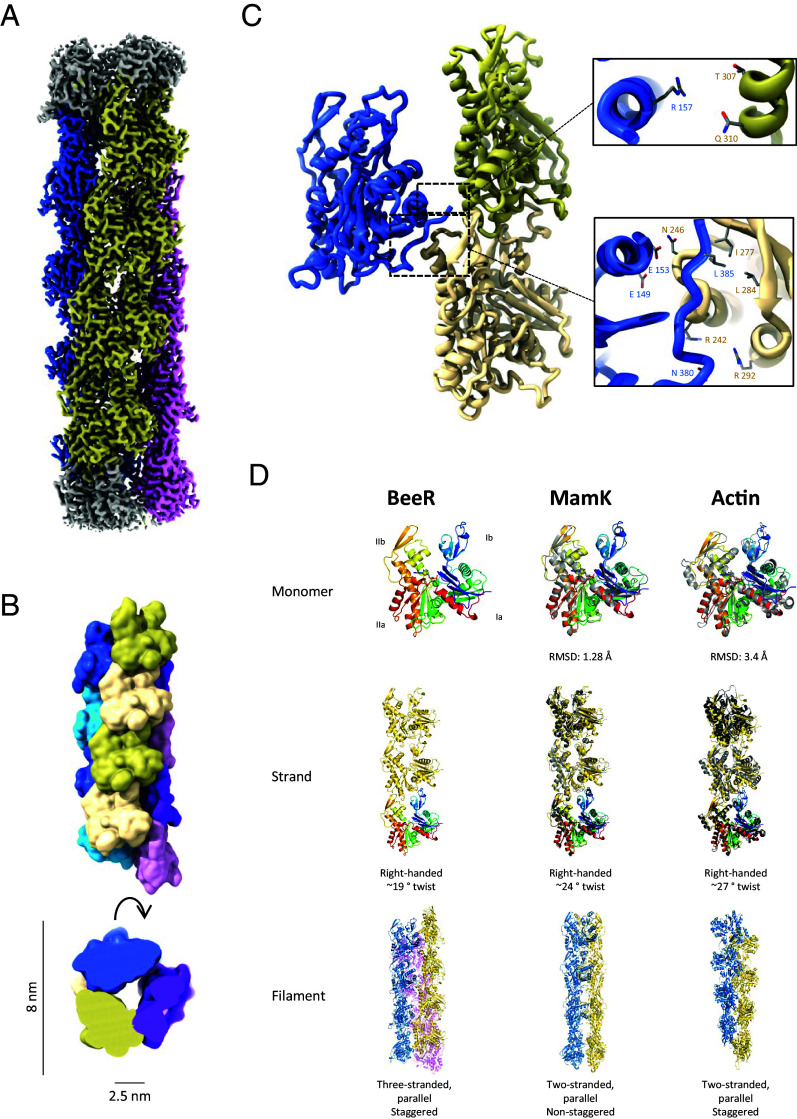
Cryo-EM structure of the BeeR tubular filament. (*A*) Cryo-EM map of the BeeR filament, to 3.1 Å resolution. The three strands are colored in dark blue, khaki, and magenta, respectively. (*B*) Surface representation of the BeeR filament atomic model, colored as in *A*, from the side and *Top*. The diameter of the filament, and internal cavity, are indicated. (*C*) Lateral contact across the strands are shown, with close-up views of the main interacting residues on the *Right*. (*D*) Comparison between BeeR and other bacterial actins. The structures of BeeR, MamK, and actin arte shown, as a monomer (*Top*), strand (*Middle*), and filament (*Bottom*). The RMSD of BeeR to MamK and Actin and the helical parameters for each are indicated.

We next used cryo-EM to determine the structure of the BeeR filament, to 3.1 Å resolution (Fig. 2*A*). This revealed that it adopts a three-stranded, parallel, right-handed, staggered filament architecture (Fig. 2*B*). The diameter of this filament, at ~80 Å, is significantly larger than most other actin-like proteins, and it includes a ~25 Å-wide cavity at its core, responsible for its rail track-like appearance by negative-stain EM.

BeeR possesses the canonical actin domains Ia, Ib, IIa, and IIb, with the nucleotide buried between domains Ib and IIb (1). It is most similar to its closest homolog of known structure, MamK (7, 8) (RMSD of 1.3 Å for aligned atoms, Fig. 2*D*). The longitudinal interface along strands is largely similar to other actin-like filaments (Fig. 2*D*), with domains Ib and IIb making extensive contacts with domains IIa of the subunit above. Its strand arrangement resembles mostly that of MamK but also actin. The N-terminal domain, which we identified as a hallmark of the BeeR family (see above and Fig. 1*D*) is not resolved in our map, supporting its intrinsically disordered nature.

The cross-strand contacts in the BeeR filament are distinct to that of other actin-like filaments. Each protomer interacts with two consecutive subunits of the adjacent strand, as shown on Fig. 2*C*. Contacts with the top subunit are minimal, with an interface area of ~35 Å^2^, and consist of a hydrogen bond network between Arg 157 of one subunit and Thr 307 and Gln 310 in the adjacent subunit. In contrast, the contact with the bottom subunit is extensive, forming a contact surface area of ~ 450 Å^2^, and consists of multiple hydrogen bonds, between Glu 149/Glu 153 and Asn 246 and between Arg 242/Arg 252 and Asn 380. In addition, Leu 385 is buried in a hydrophobic pocket of the adjacent subunit consisting of Ile 277 and Leu 284.

It is noteworthy that most of the aforementioned residues are conserved among BeeR orthologs, but not in MamK or MreB (Fig. 1*D*). This suggests that the three-stranded filament architecture is a feature of this protein family.

As mentioned above, a hallmark of the BeeR family is the presence of a predicted disordered NTD. To assess whether this NTD was involved in filament formation, we engineered a construct lacking the NTD (residues 2 to 35). As shown on (Fig. 1*D*), this construct (BeeR_ΔN_) still forms filaments, with the same overall rail-track-like morphology. However, we observed that those filaments have a very high propensity to bundle, which was not observed with the WT protein. Based on this, we propose that the NTD of BeeR acts as a “repellant,” preventing the formation of bundles that might alter their cellular function.

We emphasize that the function of the BeeR filament is not known. The diameter of its cavity (~2.5 nm) makes it unlikely that it is involved in solute or macromolecular transport. Instead, we propose that the three-stranded tubular architecture provides a much more rigid filament than other actin homologs, as supported by 2D classes (Fig. 1*C*). This likely will have an impact on its capacity to perform its function within the cell.

Nonetheless, the structure of the BeeR filament adds to our understanding of the molecular diversity of actin-like proteins, adds to the diverse structural architectures that can be formed by this family, and provides unexpected insights into the evolution of the actin fold.

## Methods Summary

The *O. terrae* BeeR homolog (UniProt accession number B1ZZL4) was overexpressed in BL21 (DE3) cells and purified by ammonium sulfate precipitation followed by size-exclusion chromatography.

Purified BeeR was applied to holey carbon grids and imaged in a Glacios TEM (Thermo Fisher) equipped with Falcon IV camera. A dataset of ~4,000 micrographs was collected and processed with CryoSPARC. The structure was determined by helical refinement, using a rise of 17.4 Å and a twist of 126.5°.

An initial atomic model of BeeR was generated with AlphaFold and refined in Phenix.

## Supplementary Material

Appendix 01 (PDF)

Movie S1. **Structure of the BeeR filament**. The three strands are shown inyellow, blue and magenta respectively, with the central cavity visible from the top.

## Data Availability

Cryo-EM map and atomic coordinates data have been deposited in EMDB and PDB [(EMD-18852 (13) and 8R2N (14)].
