# Supplementary material for: A family of bacterial actin homologs forms a three-stranded tubular structure
Source: Proc Natl Acad Sci U S A. 2025 Mar 12;122(11):e2500913122. doi: 10.1073/pnas.2500913122 (PMC11929497; doi:10.1073/pnas.2500913122)
Supplement: Supplementary file 1 — Appendix 01 (PDF) [file pnas.2500913122.sapp.pdf]

PNAS

Supporting Information for

A family of bacterial actin homologs forms a 3-stranded tubular structure.

Julien R.C. Bergeron[1]*, Shamar L. M. Lale-Farjat[1], Hanna M. Lewicka[1,3], Chloe Parry[1,3], Justin M. Kollman[2]*.

* Julien R. C. Bergeron; Justin M. Kollman
Email: julien.bergeron@kcl.ac.uk; jkoll@uw.edu

This PDF file includes:

Extended methods
Legends for Movie S1
SI References

Other supporting materials for this manuscript include the following:

Movie S1

Extended methods:

Sequence analysis:

The BeeR sequences were identified from scanning the metagenomics database[1] for MamK orthologues in non-magnetotactic bacteria, using BLAST[2]. Sequences were aligned with ClustalW[3], and a non-rooted genealogic tree was constructed with PhyML[4].

Protein expression and purification:

The *O. terrae* BeeR sequence (UniProt accession number B1ZZL4), codon-optimised for expression in *E. coli*, was cloned into pET21a. The protein was over-expressed in BL21(DE3) cells, expression was induced when the cells reach mid-log phase with 1 mM IPTG, for 16h at 20°C. Cells were then harvested at 6,000 rpm for 10 min, and lysed by sonication. Debris were then removed by centrifugation at 20,000 rpm for 45 min. BeeR was purified from the supernatant using two successive rounds of Ammonium Sulphate precipitation (15% saturation), in 25 mM KCL, 10 mM HEPES pH 7.0, 2 mM EDTA, 5 mM DTT, followed by size-exclusion chromatography using a Superdex 200 column (Cytiva), in the same buffer.

Pelleting assay:

Purified BeeR protein was concentrated to ~ 25 μM in the aforementioned buffer, but lacking the EDTA and supplemented with 5 mM $MgCl_2$. ATP or ADP were added to 5 mM, and 30 μl of the corresponding samples were spun using a benchtop centrifuge (ONiLAB) at 14,000 rpm for 20 min. The supernatant was extracted by pipetting, and the pellet was resuspended in 30 μl of the aforementioned buffer.

Negative-stain TEM:

Purified BeeR protein was diluted to ~ 0.02 mg/ml in the aforementioned buffer, ATP and $MgCl_2$ were added to 1 mM, and the resulting sample was applied to carbon-coated TEM grids. Micrographs were collected on a FEI Morgagni TEM operated at 100 kV, and equipped with a Gatan Orius camera.

Cryo-EM data collection:

Following size-exclusion chromatography, BeeR was concentrated to ~2 mg/ml, and ATP and $MgCl_2$ were added to a final concentration of 1 mM. The sample was applied to holey carbon grids, which were imaged in a Glacios TEM (Thermo Fisher) operated at 200 kV and equipped with Falcon IV camera. A dataset of ~ 4,000 micrographs was collected using EPU, with a pixel size of 1.5 Å, and a total dose of 40 e/ $Å^2$.

EM data processing:

Cryo-EM data was processed with CryoSPARC[5]. Frame alignment and CTF estimation were performed using default parameters. An initial set of ~ 1,000 particles with a box size of 512 pixels were picked manually from 10 micrographs at different defocus, and used to generate initial 2D classes. These were then used as templates for single-particle template picking, leading to a set of ~1,200,000 particles. 2D classification was employed to select the best particles (~ 900,000), which were then used for *ab-initio* structure determination. This led to a map with clear structural features that allowed to determine the helical symmetry. Following this, helical refinement was applied, using a rise of 17.4 Å and a twist of 126.5 °. This led to final map that was refined to 3.1 Å resolution.

Model building and structure analysis:

An initial atomic model of BeeR was generated with AlphaFold[6], and 36 copies were placed in the corresponding density of the EM map. ATP and Mg molecules were placed manually with Coot[7]. The obtained model was subject to real-space refinement in Phenix[8]. Map and model were displayed using ChimeraX[9] and PyMol. Interface contacts were analyzed with PISA[10].

Movie S1 (separate file).

Structure of the BeeR filament. The three strands are shown in yellow, blue and magenta respectively, with the central cavity visible from the top.

SI References

1. Wang, D. Metagenomics Databases for Bacteria. *Methods Mol Biol* **2649**, 55-67 (2023).
2. Johnson, M. et al. NCBI BLAST: a better web interface. *Nucleic Acids Res* **36**, W5-9 (2008).
3. Thompson, J.D., Higgins, D.G. & Gibson, T.J. CLUSTAL W: improving the sensitivity of progressive multiple sequence alignment through sequence weighting, position-specific gap penalties and weight matrix choice. *Nucleic Acids Res* **22**, 4673-80 (1994).
4. Guindon, S. et al. New algorithms and methods to estimate maximum-likelihood phylogenies: assessing the performance of PhyML 3.0. *Syst Biol* **59**, 307-21 (2010).
5. Punjani, A., Rubinstein, J.L., Fleet, D.J. & Brubaker, M.A. cryoSPARC: algorithms for rapid unsupervised cryo-EM structure determination. *Nature Methods* **14**, 290-296 (2017).
6. Jumper, J. et al. Highly accurate protein structure prediction with AlphaFold. *Nature* **596**, 583-589 (2021).
7. Emsley, P. & Cowtan, K. Coot: model-building tools for molecular graphics. *Acta Crystallographica Section D* **60**, 2126-2132 (2004).
8. Afonine, P.V. et al. Real-space refinement in PHENIX for cryo-EM and crystallography. *Acta Crystallogr D Struct Biol* **74**, 531-544 (2018).
9. Pettersen, E.F. et al. UCSF ChimeraX: Structure visualization for researchers, educators, and developers. *Protein Sci* **30**, 70-82 (2021).
10. Krissinel, E. Stock-based detection of protein oligomeric states in jsPISA. *Nucleic Acids Research* **43**, W314-W319 (2015).
